# Heterogeneous worker multi-functionality and efficiency in dual resource constrained manufacturing lines: an assessment by simulation

**DOI:** 10.1007/s12063-023-00371-2

**Published:** 2023-04-19

**Authors:** Federica Costa, Matthias Thürer, Alberto Portioli-Staudacher

**Affiliations:** 1 Department of Management, Economics and Industrial Engineering, Politecnico di Milano, Via Lambruschini 4/b, Milano, 20156 Italy; 2 Chair of Factory Planning and Intralogistics, Chemnitz University of Technology, Erfenschlager Straße 73, Chemnitz, 09125 Germany

**Keywords:** Worker assignment, Heterogeneous labor force, Dual resource constrained shop, Manufacturing line balancing, Labor flexibility, Labor efficiency

## Abstract

Flexibility is a main mean to create resilient supply chains. The most flexible resources are often human resources but creating high, homogenous skill levels is not cost efficient. Heterogenous labour provides an alternative. The literature on Dual Resource Constrained (DRC) shops modelled heterogeneous labour with multi-functionality and efficiency matrices that indicate if a worker can perform at a station and according to which level of efficiency. However, this literature typically considered these matrices as a given environmental factor rather than a factor under firm control. Consequently, it typically neglected literature that can be used to guide matrix design. In response, this study draws on the literature on unbalanced lines to test eight different matrices to guide worker training. Discrete event simulation is then used to evaluate their performance in a DRC pure flow shop with high variability in demand and processing times. Results demonstrate that the matrix design impacts performance and that an unbalanced design has the potential to improve performance compared to a balanced design and full multi-functionality. Specifically, the bowl configuration shows much promise, which further confirms the “bowl phenomenon”. However, performance gains are dependent on the combination of When, Where and Who rules used to guide the worker assignment decision. So, the decision on worker training is also contingent on the decision which worker assignment rule to use. Findings have important implications for research and practice, providing guidance on how to design more resilient shops and supply chains.

## Introduction

Flexible capacity is widely recognized as a mean to accommodate uncertain market demand (e.g. Altendorfer et al. 2020; Chou et al. 2016; Fan et al. 2022). This potential becomes even more important with the increase of uncertainty caused by changes in consumer behaviour patterns witnessed recently (Ardolino et al. 2022). These changes together with shortages in manufacturing resources (Ivanov and Dolgui 2022) lead to systemwide disruptions that effect whole supply chains. Disruptions, such as those caused by COVID-19, create ripple and bullwhip effects that create uncertainties in demand within and across whole industries (e.g. KEK et al. 2022). In this context, flexibility is a main mean for firms to build resilience capability into their systems to survive and grow (Piprani et al. 2022; Badhotiya et al. 2022), specifically reconfigurability (Pansare and Yadav 2022).

The most flexible resource on the shop floor is often the worker; in practice workers are often flexible and can be shifted from one station to another (Erhard 2019; Hopp and Oyen 2004) reconfiguring systems at short notice. While it is obvious that the best performance can be obtained if workers are homogenous in terms of high levels of multi-functionality and efficiency, it is often not desirable to rely on such a highly skilled workforce. First, there is a trade-off between efficiency gains through standardization and flexibility (Assad Neto et al. 2021). Second, employing skilled workers is generally limited in real-world scenarios because of high wages in high-cost environments, such as developed economies (Mirzaei et al. 2021), and the absence of trained labour in less developed contexts (Zheng and Wang 2016).

Using a heterogeneous (Kim and Nembhard 2013; Lian et al. 2018) instead of a homogenous workforce can provide significant reduction in training needs and costs (Brusco and Johns 1998). If the workforces is heterogenous, then managers need to decide on the most efficient assignments of machines and workers, while keeping worker workload balanced (Vital-Soto et al. 2023). This decision has a major impact on efficiency and resilience. But in addition, managers also need to decide on worker training, since this determines the assignments that can be realized. Compared to a specialized system where specific tasks (or operations) can only be handled by one worker, cross-training creates a flexible system through overlapping skills that enable tasks to be processed by alternative workers (Bokhorst and Gaalman 2009). This enabling may occur in terms of so-called *multi-functionality*, which is the number of tasks a worker can execute, and in terms of *efficiency*, which is the rate at which a task can be executed.

There exists a broad literature on heterogeneous labour, specifically in the context of Dual Resource Constraint (DRC) shops, where workers are considered a constraining resource that can be transferred across a given set of stations (Xu et al. 2011; Araz and Salum 2010). However, this literature considers worker skills to be an environmental variable, and performance of, for example, labour assignment rules are evaluated taking this environmental variable into account. Meanwhile, there also exists a broad literature considering the DRC scheduling problem. But only very few studies have considered heterogeneous worker. For example, Liu et al. (2022) considered processing time reductions resulting from increased experience at the job level and motivation at the workforce level, Dunke and Nickel (2022) combined a multi-method approach to solve the DRC job shop scheduling problem with data uncertainty and worker efficiency variation over time, Lei and Guo (2014) considered a DRC interval job shop with heterogeneous workers considering environmental objectives, while Zheng and Sui (2019) considered the same problem but with the objective to minimize energy consumption. Geurtsen et al. (2023) provides an overview of the literature on resource scheduling that consider resources that reduce the processing time (speed-up resource). A major shortcoming of this scheduling literature is its restriction to deterministic contexts, i.e. a solution for a given set of jobs is obtained. But in practice, many shops experience stochastic contexts, specifically shops that operate as make-to-order shops, and were jobs can arrive at any moment in time. This makes the use of scheduling questionable. Moreover, again the worker skill levels are seen as environmental factors not under the influence of management. This means, some standard levels of heterogeneity are tested, and no specific matrices designed with the objective to improve performance.

To the best of our knowledge, the only two existing study that test different multi-functionality and efficiency matrices for heterogeneous labour with the objective to guide worker training and eventually improve performance are Brusco and Johns (1998) and Bokhorst et al. (2004a, b). Brusco and Johns (1998) tested different cross-training configurations, showing results that asymmetric efficiency matrices always outperformed symmetric matrices, which reinforces the importance of matrix design for performance improvement. However, these results are based on integer programming models and cost calculations. The actual operational impact of different labour multi-functionality and efficiency matrices on performance remains unknown. Bokhorst et al. (2004a, b) found that in parallel and job shop structures an equal multi-functionality and an equal machine coverage are important for achieving an optimal mean flow, whilst serial structures require more attention and unequal multi-functionality. Our study extends Brusco and Johns (1998) and Bokhorst et al. (2004a, b) by considering heterogeneous efficiencies and by drawing on the wider Operations Management literature to identify rules for matrix design. For example, the bowl phenomenon (McNamara et al. 2016) would suggest an increase in capacity at central stations through either increasing worker efficiency or multi-functionality at these stations. However, none of the matrices presented in the DRC literature creates this bowl shape. In this study, we therefore explore the broader Operations Management literature to identify different matrix designs for a DRC shop with heterogeneous labour to answer the following research question that motivates the study “*What is the best design for multi-functionality and efficiency matrices in a high variety make-to-order flow shop with heterogeneous labour?”*

We then use simulation to assess performance of the different designs in a DRC pure flow shop with high variability in demand and processing times. From a theoretical perspective, this integrates different related but so far unconnected streams of literature. It also reframes the DRC problem as a design problem, which is important when shops and entire supply chains are rebuild for resilience. From a practical perspective, we seek to provide guidance to managers on worker training in this type of shop, which is a common shop type, e.g. for companies that focus on producing prototypes and making small runs, e.g. of 1 to 4 units, sometimes referred to as “one offs” (Rossini et al. 2019; Thürer et al. 2015).

The remainder of this paper is structured as follows. In Section 2, we review the relevant DRC literature on heterogeneous labour in terms of multi-functionality and efficiency, and the literature on unbalanced lines. The simulation model used to evaluate performance is then described in Section 3 together with the design rules that emerged from the literature and the associated training matrices to be considered in our study. Finally, the results are presented, discussed, and analysed in Section 4 before conclusions are presented in Section 5, where managerial implications, limitations, and future research directions are also outlined.

## Literature review

This study links into two streams of literature. First, the DRC literature that considers a heterogeneous workforce in terms of multi-functionality and efficiency. Second, the literature on unbalanced lines. Both are discussed in Sections 2.1 and 2.2 respectively.

### Heterogeneous labour in DRC shops

This section reviews the literature on heterogeneous multi-functionality and heterogeneous efficiency. Note that we only focus on literature in stochastic environments. This excludes most of the literature on advanced scheduling techniques (e.g. Lei and Guo 2014, 2015; Li et al. 2016; Zheng and Wang 2016; Zhang et al. 2017; Li et al. 2016; Liu et al. 2022; Dunke and Nickel 2022; Gong et al. 2018), which presupposes that demand and capacity availability are known in advance and therefore deterministic. Most literature on stochastic environments approaches the problem of worker assignment in DRC shops via the greedy heuristic of When?, Who?, and Where?. The When rule just triggers the assignment decision and needs to be executed even for more advanced assignment procedures. Meanwhile, the Who rule is only meaningful if there is more than one worker available, while the Where rule is only meaningful if there is more than one station in need of a worker. Given our stochastic environment, it is very unlikely that both situations occur simultaneously, except for low load periods. We therefore consider that the focus on studies that use above greedy heuristic is justified.

#### Heterogeneous multi-functionality

Labour multi-functionality has been defined as the number of different departments or stations at which a worker can perform operations (Costa and Portioli-Staudacher 2021; Fry et al. 1995; Park and Bobrowski 1989). Nelson (1967) was one of the first to introduce the concept of labour multi-functionality, but then only considered a completely inflexible scenario, where each worker can work at only one station, and a completely flexible scenario, where each worker can work at any station. These scenarios were extended by two scenarios where worker can work at two or three stations in a five station DRC job shop by Park and Bobrowski (1989), while Park (1991) also included the scenario where a worker can work at four stations. A major conclusion from these earlier studies is that most of the benefits associated with worker multi-functionality can be realized without extreme high multi-functionality levels or full multi-functionality with all workers cross-trained on all machines (e.g. Fry et al. 1995; Campbell 1999). Fry et al. (1995) consequently only considered scenarios where workers can work at 1, 2 and 3 stations.

Felan and Fry (2001) later suggest that it is better to have a mix of workers with no multi-functionality and some workers with very high multi-functionality rather than all workers with equal multi-functionality. Meanwhile, Bokhorst et al. (2004a, b), Slomp et al. (2005) and Yue et al. (2008) incorporate the concept of chaining (Jordan and Graves 1995; Iravani et al. 2007) in cross-training configurations that link workers to machines. For example, Yue et al. (2008) showed that a minimum level of heterogeneous multi-functionality is desired and that long chain, where all workers have overlapping capabilities, is in all situations better than several short chains, where capabilities do not always overlap.

#### Heterogeneous efficiency

Labour efficiency is the level of service rate at which a worker can work at a station (Bobrowski and Park 1993). If a worker is assigned to a new station, then a productivity loss is likely to occur. The concept of multi-functionality does not consider this effect; rather workers are assumed to either have the maximum level of efficiency or to be unable to work at a station (see e.g. Thürer et al. 2019). This would be an ideal situation where operators are perfectly interchangeable without productivity losses due to different sets of skills or experience, an assumption first questioned by Bobrowski and Park (1993). Bobrowski and Park (1993) used three heterogeneous labour efficiency matrices: (i) each worker is fully efficient at one station but only realizes 95% of its service rate at the next four downstream stations and 85% at the remaining stations; (ii) the overall aggregate worker efficiency is decreasing, and the worker realizes 95% of its service rate at the next downstream station, 90% at the three following downstream stations and 80% at the remaining stations; and (iii) workers are divided in two groups, a senior group made up of five workers and a junior group made up of the four remaining workers, and junior workers are able to operate stations with an efficiency that can at best match the worst of the senior worker’s efficiency for those stations. Results in Bobrowski and Park (1993) show that efficiency is likely to dominate the labour assignment rules in contexts with heterogeneous efficiencies. This result was later confirmed by Bokhorst et al. (2004a, b), who found that heterogeneity outperforms homogeneity because the former increases the effectiveness of the Who rule. A similar finding was obtained by Thürer et al. (2020). While this result is important in terms of assignment rules, no real information on the impact of differences in the efficiency matrices could be gained. As expected, reducing worker’s average efficiency across stations results in worse performance.

The study by Bobrowski and Park (1993) was later extended by Malhotra and Kher (1994) who modelled three different heterogeneous efficiency matrices. In the first, workers where subdivided according to efficiency, e.g. worker A 100% efficient at all stations, worker B 95% efficient at all station and so on. In the second, stations where subdivided according to efficiency, i.e. all workers are 100% efficient at station 1, 95% at station 2 etc. The third resembles the matrices in Bobrowski and Park (1993). According to Malhotra and Kher (1994), rather than having both heterogeneous workers and stations (the third matrix) it is better to train workers in order to have some stations that always work faster, for example the first machine, or having some workers completely interchangeable and some others less efficient; the latter is similar to the senior and junior worker concept proposed by Bobrowski and Park (1993) and overlaps with the results in Felan and Fry (2001) on labour multi-functionality.

### Theoretical background on unbalanced lines

Line balancing is an important problem in the Operations Management literature and has consequently received broad research attention (see the reviews by Boysen et al. 2008; Battaïa and Dolgui 2013). Line balancing may hereby focus on an equal distribution of work content across the stations of a line (e.g. Parvin et al. 2012), or on an equal distribution of capacity. The focus of this study is on the latter. Balanced lines – where service time means of stations are all equal – generally lead to best performance (Salveson 1955), but some studies also recognized the importance of unbalanced lines (i.e. Lau 1992; Shaaban and McNamara 2009), since they can perform equally well as balanced lines in certain contexts (McNamara, Shaaban, and Hudson 2016) but require less capacity. Unbalanced line hereby means that stations do not operate at the same service rate (McNamara et al. 2016), i.e. resulting in some stations being faster in processing a job than some other stations. This is equivalent to the impact of a heterogeneous labour force discussed in the context of DRC shops above, since the service rate is a result of the capacity, which itself is realized by the machine and the worker in a DRC shop. Machine and worker can constrain the service rate, being the impact of the worker typically more pronounced in less automated process, such as in high variety contexts.

One of the most important discoveries in the area of unbalanced lines was the “bowl phenomenon”. The bowl phenomenon was first observed by Hillier and Boling (1967), who demonstrated that the output of a serial production line with up to 4 stations with exponentially distributed processing times and limited inter-station buffers could be increased by unbalancing the line: faster stations should be positioned in the middle of the line. Hillier and Boling (1967, 1979) further argued that a symmetrical bowl shape was the optimal for all line length, while El-Rayah’s (1979) findings supported the bowl phenomenon for lines having up to 12 stations, and Pike and Martin’s (1994) for lines with 30 stations.

More recently, there has been a resurgence of interest in the bowl phenomenon (e.g. Lopes et al. 2020; McNamara et al. 2016; Romero-Silva and Shaaban 2019) and the literature explored the bowl phenomenon considering different aspects, such as the effect of variance of the operations times, the effect of the buffer capacity and the effect of considering unreliable lines. In these contexts, the bowl phenomenon may not always lead to the expected improvements (e.g. Romero-Silva et al. 2021). In general, the bowl phenomenon is argued to be more important in context with limited inventory buffer size. In this study, we will assess whether it has an impact in DRC shops where labour is heterogeneous.

## Simulation

We model companies whose production follows a dominant sequence flow, i.e. a production line. A simulation model of a pure flow shop has been implemented in Simio, a specialized software for discrete event simulation (Rossini and Portioli-Staudacher 2018; Kundu et al. 2018). A stylized standard model of a pure flow shop will be used in this study to avoid interactions that may otherwise interfere with our understanding of the main experimental factors (Portioli-Staudacher et al. 2020). While any individual flow shop in practice will differ in many aspects from our stylized environment, the model used in this study captures the job and shop characteristics of high variety make-to-order flow shops, i.e. high processing time variability,

### Overview of modelled shop and job characteristics

We have kept our flow shop relatively small since this allows causal factors to be identified more easily. Small systems provide a better insight into the role of operating variables and, in practice, large systems can often be decomposed into several smaller systems (Bokhorst et al. 2004a, b). The shop has five stations with unlimited buffers, which also allows for comparison with previous DRC studies (Davis et al. 2009; Park and Bobrowski 1989; Park 1991). Each station has two identical machines. We choose a staffing level of 50% (e.g. Felan et al. 1993). In other words, there are five workers that can be shifted across the ten machines and worker’s availability constrains the capacity. Only if a worker is assigned to a machine, the capacity of the machine is realized and orders can be processed. As in previous DRC research, we consider machine capacity to be constant and we instead focus on different levels of labour capacity (Thürer et al. 2019; Thürer et al. 2020).

As in most previous DRC studies, travel time is considered negligible. Operation processing times follow a 2-Erlang distribution with a mean equal to 60 time units (e.g. Thürer et al. 2020; Bokhorst et al. 2004a, b). Set-up times are considered sequence independent and consequently part of the operation processing time. The inter-arrival time of jobs follows an exponential distribution with a $\lambda$ that ensures that workers are, on average, occupied for 90% of their time if a worker is chosen at random among the set of available workers. Due dates are set exogenously by adding a random allowance factor, uniformly distributed between 1600 and 1900 time units, to the job entry time. The minimum is set considering the maximum processing time and the routing length of jobs that is equal to the number of stations, five. The maximum was set such that the percentage tardy is neither too high nor too low. The percentage tardy should not be too high to avoid certain adverse effects, since rules that reduce the variance of lateness across jobs may even lead to an increase in the percentage tardy when due date allowances are too tight on average. The percentage tardy should not be too low to avoid our results being affected by incidental effects, as very few jobs would be responsible for the performance of the shop. Finally, the first come first served rule is used for priority dispatching.

### Matrix design

The following design rules could be identified from the literature:

- *Design Rules from the Literature on Heterogeneous Multi-Functionality *(Section 2.1.1): High levels of multi-functionality are not required. Rather, a small number of highly skilled workers (being all other workers lower skilled) is preferred to a scenario where all workers are equally skilled. Long overlapping chains are preferred.
- *Design Rules from the Literature on Heterogeneous Efficiency *(Section 2.1.2): Efficiency matrices should focus on equipment heterogeneity, i.e. the first station works faster than the others, or labour heterogeneity, i.e. some workers are more proficient on all machines then the others. Asymmetric chaining configuration, such as the CT4 long chain configuration in Brusco and Johns (1998), are preferred to symmetric matrix configurations, such as the CT1 small chain configuration in Brusco and Johns (1998).
- *Design Rules from the Literature on Unbalanced Lines *(Section 2.2): Placing faster workers at middle stations (bowl shape) can lead to lower throughput times. Placing faster workers at stations either at the beginning or the end of a line (inverted bowl) can lead to improvements in total completion time. Placing workers from slow to fast following the workflow (ascending order) can reduce work-in-process.

While these rules were obtained across differing contexts, and may not be directly comparable and transferable, they provide important indications on which matrix designs to consider in our study. Given these rules, eight different matrices for multi-functionality will be tested in this study as follows:

1. *Bowl*: more workers are trained for central stations;
2. *Inverted bowl or bell*: less workers are trained for central stations;
3. *Downstream increasing (DSI) or monotone ascending*: less workers are trained for upstream stations;
4. *Upstream increasing (USI) or monotone descending*: more workers are trained for downstream stations;
5. *Long chain*: all workers are involved in a long chain of skill overlaps with the average number of skills that overlaps equal to 2 (as in Yue et al. 2008);
6. *Complete Long Chain*: all workers are involved in a chain of skill overlaps with the average number of skills that overlaps equal to 2 (as in Yue et al. 2008) and the long chain is completed and closed with the first worker being able to operate on the fifth station (Inman et al. 2004);
7. *Small chain*: two short chains with two workers being involved in each chain of skill overlaps with the average number of skills that overlap equal to 2 (as in Yue et al. 2008); and,
8. *Full multi-functionality (Full)*: all workers can work at all stations. This homogeneous scenario is considered to provide a baseline for comparison with the previous matrices.

In terms of efficiency matrices, we consider workers to be able to work at each station, however at different proficiency. For this reason, we only consider small, long chain and complete long chain. The different matrices for multi-functionality and efficiency considered in this study are summarized in Table 1, which indicates the stations at which a worker can work for the multi-functionality scenarios, and at which a worker realizes 100% efficiency for the efficiency scenarios. At other stations, workers can either not work (multi-functionality scenarios) or work at reduced efficiency. The efficiency loss $\alpha$ of each worker is modelled at three levels: 10%, 20% and 30%, meaning that the worker needs *p*/(1-$\alpha$) time units to finish the job, where *p* is the processing time if the job was processed by a fully efficient worker. Since the average efficiency of the workers is reduced, we adjust the inter-arrival rate of jobs as in e.g. Thürer et al. (2019) to ensure comparable worker utilization levels across experiments. This adjustment is realized by simply multiplying the inter-arrival rate by the average level of efficiency.

**Table 1:**
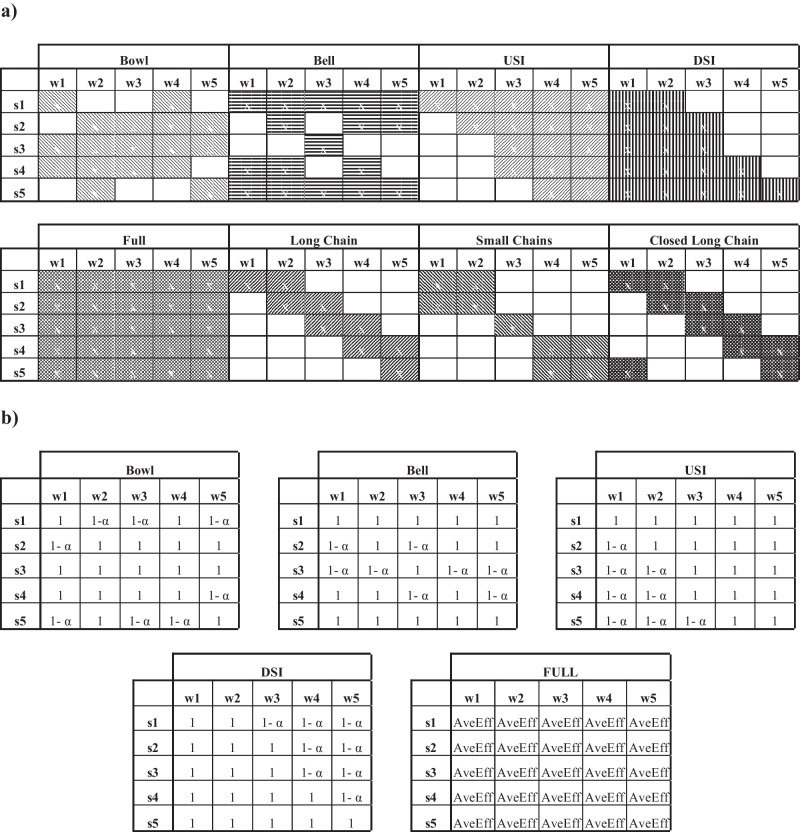
Summary of multi-functionality **a**) matrices where it is shown whether a worker can work on a station and efficiency **b**) matrices where it is shown the efficiency of each worker on the different stations with α being the efficiency loss. In the Full configuration for Efficiency, workers can work at all stations with the same level of efficiency – AveEff – that is equal to the average efficiency of the other matrices (Bowl-Bell-USI-DSI)

### Labour assignment rules

There are three major worker assignment decisions. First, *when* should the worker assignment decision be executed (i.e. the When Rule)? Second, for the case that there are multiple stations without a worker and therefore available to receive workers, *where* should a worker go (i.e. the Where Rule)? And third, for the case that more than one worker is not assigned to a station and is therefore available, *who* should be assigned to the station (i.e. the Who Rule)?

In this study, two When Rules are considered: (i) centralized, where a worker is eligible for transfer after each job completion; and, (ii) decentralized (exhaustive), where a worker is eligible for transfer once the queue of the current station is empty (or exhausted). We also consider three Where Rules: (i) maximum number of jobs in queue (Max Jobs), where a worker is transferred to the station with the longest queue measured in terms of number of jobs; (ii) earliest due date (EDD), where a worker is transferred to the station with the queue that contains the job with the most urgent due date; and, (iii) maximum efficiency (Max Eff), where a worker is transferred to the station where it has the maximum efficiency. For all three, the selected station may be the current station or a station without a worker. The Max Jobs rule was included as a standard rule that has been widely applied in previous research. The EDD rule was included due to its good performance in Jensen (2000), while the Max Eff rule should be considered because we consider a workforce that is heterogeneous in proficiency. Other rules, such as the shortest processing time rule were not considered since they did not lead to performance improvements in previous studies (see, e.g. Park and Bobrowski 1989). Finally, we also consider two Who Rules: (i) the Random rule, where the worker will be chosen randomly; and, (ii) the Max Eff rule, where the most efficient worker will be chosen from the set of available workers.

### Experimental design and performance measures

The experimental factors are summarized in Table 2, for multi-functionality and Table 3, for efficiency. A full factorial design with 32 (2x2x8) scenarios for multi-functionality and 180 (2x3x2x5x3) scenarios for efficiency was used. Each scenario was replicated 100 times. Results were collected over 1.000.000 time units following a warm-up period of 400.000 time units. These parameters allowed us to obtain stable results while keeping the simulation runtime to a reasonable level. Three main performances measures are used to assess both workload balancing and delivery performance: (i) the lead time (i.e. the time when a job is completed minus the time when it entered the shop); (ii) the percentage of tardy jobs; and, (iii) the mean tardiness – that is *T*_*j*_=max (0,*L*_*j*_), with *L*_*j*_ being the lateness of job *j* (i.e. the actual delivery date minus the due date of job *j*). We do not explicitly consider training costs since these costs are idiosyncratic to each company. Similar holds for the revenue increase realized through the different matrices, to which training costs would have to be compared. We therefore do not consider cost, but companies can use the operational results to estimate costs applying their idiosyncratic cost and revenue factors.

**Table 2:** Experimental setting for Multi-functionality

Factors	Levels
When Rule	Decentralized and Centralized
Where Rule	EDD (earliest due date) and Max Jobs (maximum number of jobs)
Who Rule	Random
Matrices	Bowl – Bell – DSI – USI – FULL – Small Chain – Long Chain –Complete Long Chain

**Table 3:** Experimental setting for Efficiency

Factors	Levels
When Rule	Decentralized and Centralized
Where Rule	EDD (earliest due date), Max Jobs (maximum number of jobs) and Max Eff
Who Rule	Random and Max Eff
Matrices	Bowl – Bell – DSI – USI - FULL
Efficiency Loss (α)	10%, 20%, 30%

## Results

Statistical analysis of our results was first conducted using an ANOVA (Analysis of Variance). Given our experimental set-up, we conducted two ANOVA’s, one for multi-functionality and one for efficiency. For the efficiency related experiments, efficiency loss was treated as blocking factor, which is commonly used to model known sources of variance. Results are not presented given space restrictions. Most main effects, two-way interactions, three-way interactions, and the four-way interaction (for efficiency experiments) were shown to be statistically significant at $\alpha =$ 0.05. Detailed results will be presented next to explore these performance differences. Section 4.1 first focusses on multi-functionality. Section 4.2 then presents the results for efficiency matrices. Finally, a discussion of results is presented in Section 4.3.

### Performance assessment for multi-functionality

**Fig. 1:**
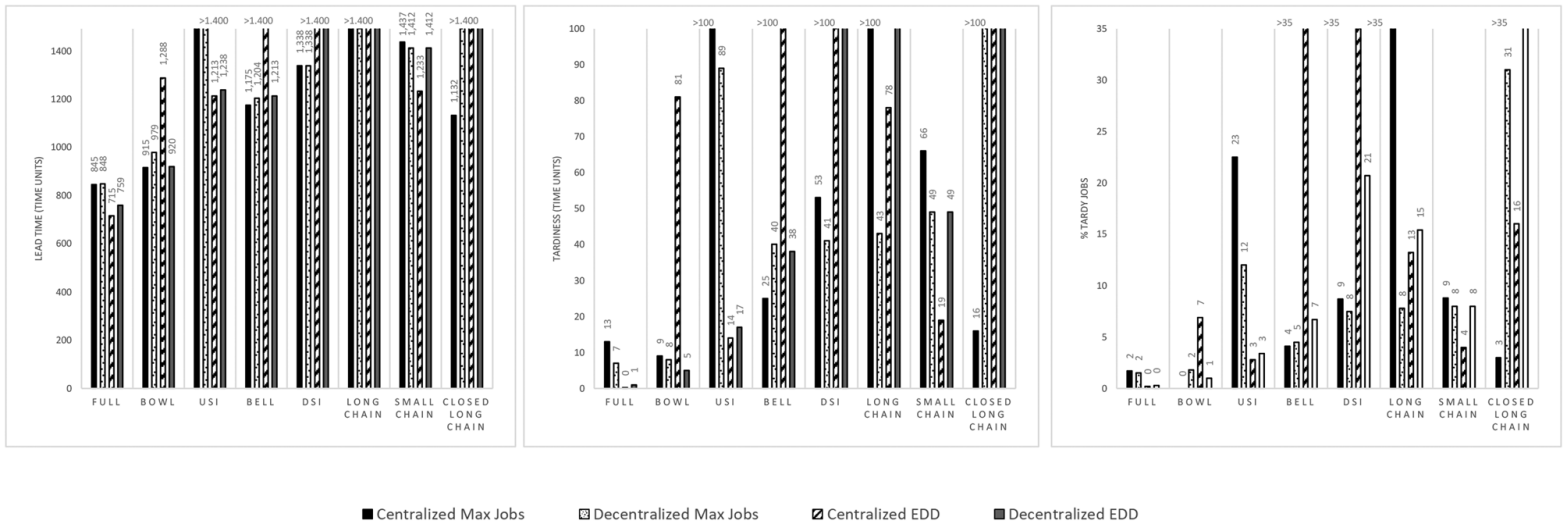
Lead time, Tardiness, and %Tardy Jobs for Multi-functionality

The results obtained for the experiments considering the eight different matrix configurations for multi-functionality are summarized in Fig. 1. We structured the presentation of results along the performance of the different matrix configurations, given that this is the focus of our study. The performance of each configuration is highly dependent on the assignment rules applied. The impact of assignment rules in isolation will be discussed further below.

As somewhat expected, Full results in best performance. However, Bowl realizes comparable performances, especially for Max Jobs (with both Centralized and Decentralized When Rule) and EDD (with Decentralized When Rule). The USI configuration realizes good performance with EDD and Centralized When Rule. With USI only 3 workers can work at station 4 and only 2 workers can work at station 5. EDD incentivizes the transfer of worker to downstream stations, which partly overcomes this shortage specifically if a Centralized When Rule is used, and workers can move more frequently. This also explains why Bell and DSI show poor performances with EDD and a Centralized When Rule. In general, USI performs well with EDD and poorly with Max Jobs, while DSI performs well with Max Jobs and poorly with EDD. Bell performs well with Max Jobs and a Centralized When Rule.

Meanwhile, Small Chain performs better than Long Chain and Closed Long Chain. Long Chain shows very poor performance with Max Jobs and a Centralized When Rule. Under this setting Long Chain leads to larger queues at downstream stations, especially in front of station 5, as shown in Table 4.

**Table 4:** Queue length (QL) in job’s unit for Long Chain, Closed Long Chain and Small Chain

	QL1	QL2	QL3	QL4	QL5	QL1	QL2	QL3	QL4	QL5
Long Chain	**Centralized Max Jobs**					**Decentralized Max Jobs**				
	1,23	4,01	6,71	10,92	18,12	2.31	1,44	2,32	4,13	6,75
Complete Long Chain	2,88	2,34	2,23	2,17	2,13	6,58	14,9	5,80	6,23	6,53
Small Chain	3,54	3,18	5,14	2,25	2,03	3,94	2,90	4,63	2,38	2,19
Long Chain	**Centralized EDD**					**Decentralized EDD**				
	1,63	3,87	3,85	4,66	6,13	2,07	2,19	3,6	5,23	8,11
Complete Long Chain	17,39	1,23	1,01	0,85	0,49	7,64	12,87	11,04	9,18	6,17
Small Chain	4,42	0,56	4,48	2,80	0,41	3,94	2,90	4,63	2,38	2,19

These larger queues lead to workers 4 and 5 having a higher utilization compared to other workers when Long Chain is used. This is due to station 4 and 5 only realizing a 50% utilization when worker 4 is at station 3. It only occurs with Max Jobs since the likelihood that worker 4 is at station 3 is higher compared to EDD. Closing the Long Chain improves performance only when Max Jobs and a Centralized When Rule is used, because at station 5 the utilization is increased, and consequently there are no large queues at downstream stations. Meanwhile, if the Max Jobs Where Rule is combined with a Decentralized When Rule, the Long Chain and Small Chain matrices have comparable performances. These findings are not in line with Yue et al. (2008), where long chains – in which all workers have overlapping capabilities – perform better than small chains, in a job shop with a Max Jobs Where Rule and a Centralized When Rule. This will be discussed further in Section 4.3 below.

Finally, and in terms of labour assignment rule, we observe that the Decentralized (exhaustive) outperforms the Centralized When rule with Max Jobs, except for Bell. For Bell only worker 2 and 4 can work at station 2 and 4, while worker 3 cannot. A Centralized When Rule increases the chances that a worker is shifted. Meanwhile, a EDD Where Rule performs better than Max Jobs for Full, Bowl and USI if the Decentralized When Rule is applied and for Small Chain, Long Chain, USI and Full if a Centralized When Rule is applied.

### Performance assessment for efficiency

**Fig. 2:**
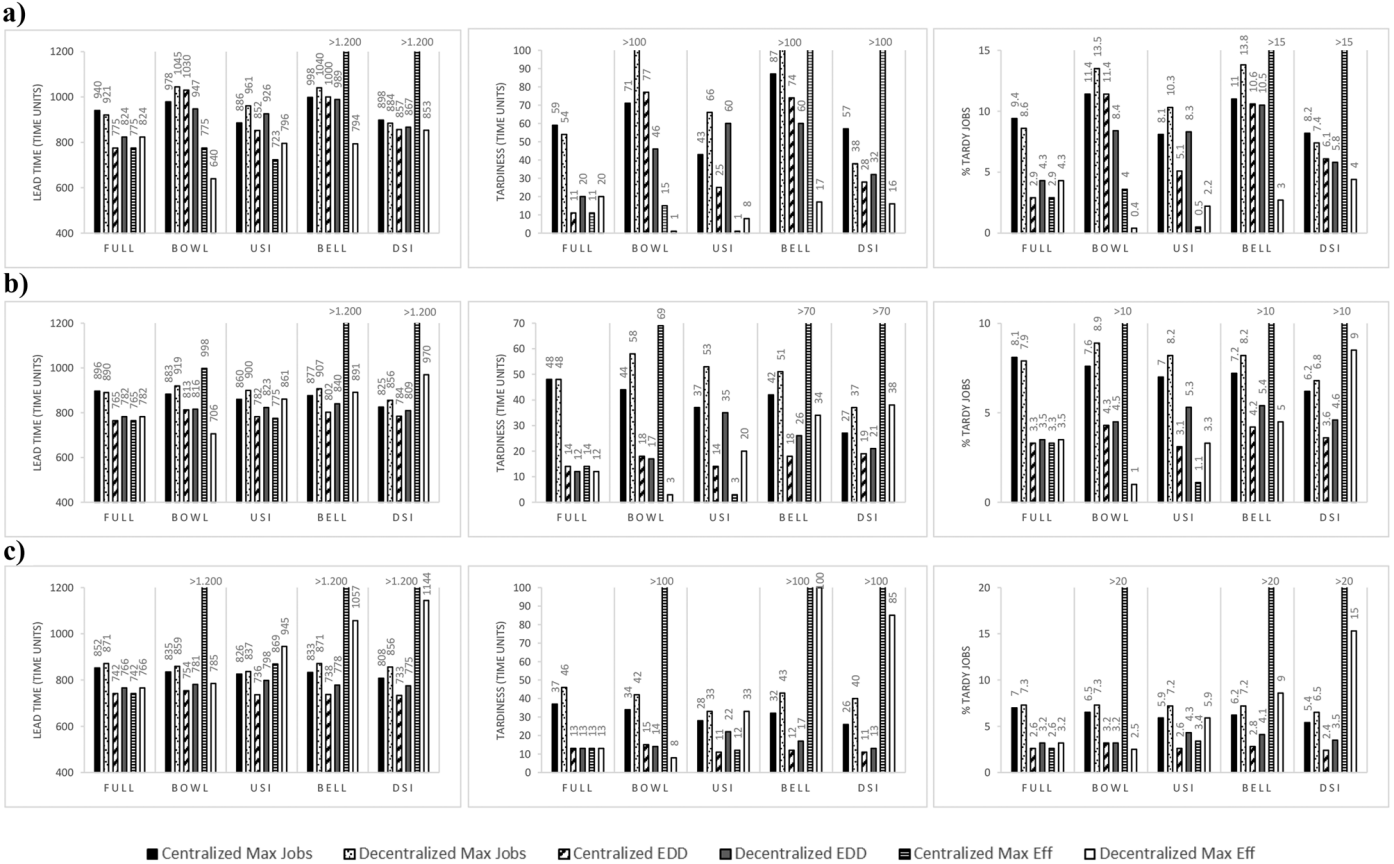
Lead time, Tardiness, and %Tardy Jobs for Efficiency with Max Eff Who Rule for efficiency loss: (**a**) 0,3; (**b**) 0,2; and, (**c**) 0,1

The results for the experiments considering the five different matrix configurations for efficiency are summarized in Fig. 2 for the Max Eff Who Rule. The results for the Random Who rule are provided in an Appendix since this factor was not significant. Note that results across the three different levels of efficiency loss $\alpha$ (0,3; 0,2 and 0,1) are not directly comparable since an adjustment in inter-arrival time was required to ensure equal worker utilization. Differences across $\alpha$ are therefore not discussed, which is justified by the focus of our study, which is on the impact of matrices for each setting of $\alpha$. We again present results along the performance of the different matrix configurations, and the impact of assignment rules in isolation further below.

For all setting of $\alpha$, Bowl performs better than Full if a Max Eff Where Rule and a Decentralized When Rule is used. This confirms the findings in the literature that heterogeneous configurations are better than homogeneous configurations. Differences compared to the results for multi-functionality can be explained by inter-arrival times being adjusted to account for differences in efficiencies across matrices. So, in a sense the comparison to Full is fairer for efficiency than for multi-functionality. The Bowl, Bell and DSI configuration perform poorly if a Centralized When Rule is used. This is explained by the fact that with Max Eff, workers tend to stay where they are more efficient, and a Centralized When Rule aggravates this effect. Meanwhile, USI works well with EDD, as in the multi-functionality scenario. DSI has workers that have an efficiency loss at upstream stations, however, when efficiency loss is low, DSI performs similarly to USI. This is also the reason why with Max Jobs, DSI outperforms Full, especially with a Decentralized When Rule while Bowl and Bell perform poorly.

Finally, in terms of worker assignment rule, we observe that with Max Jobs and EDD Where Rule, a Centralized When Rule outperforms a Decentralized When Rule for each configuration for low levels of $\alpha$. But when $\alpha$ is high, Decentralized is better for DSI and Full (with Max Jobs) and for Bowl and Bell (with EDD). In general, EDD outperforms Max Jobs and the Max Eff Where rule outperforms EDD, especially when $\alpha$ is high. As shown in previous literature, if labour is heterogeneous in terms of proficiency, then efficiency considerations tend to dominate the choice of assignment rule.

#### Robustness analysis

**Fig. 3:**
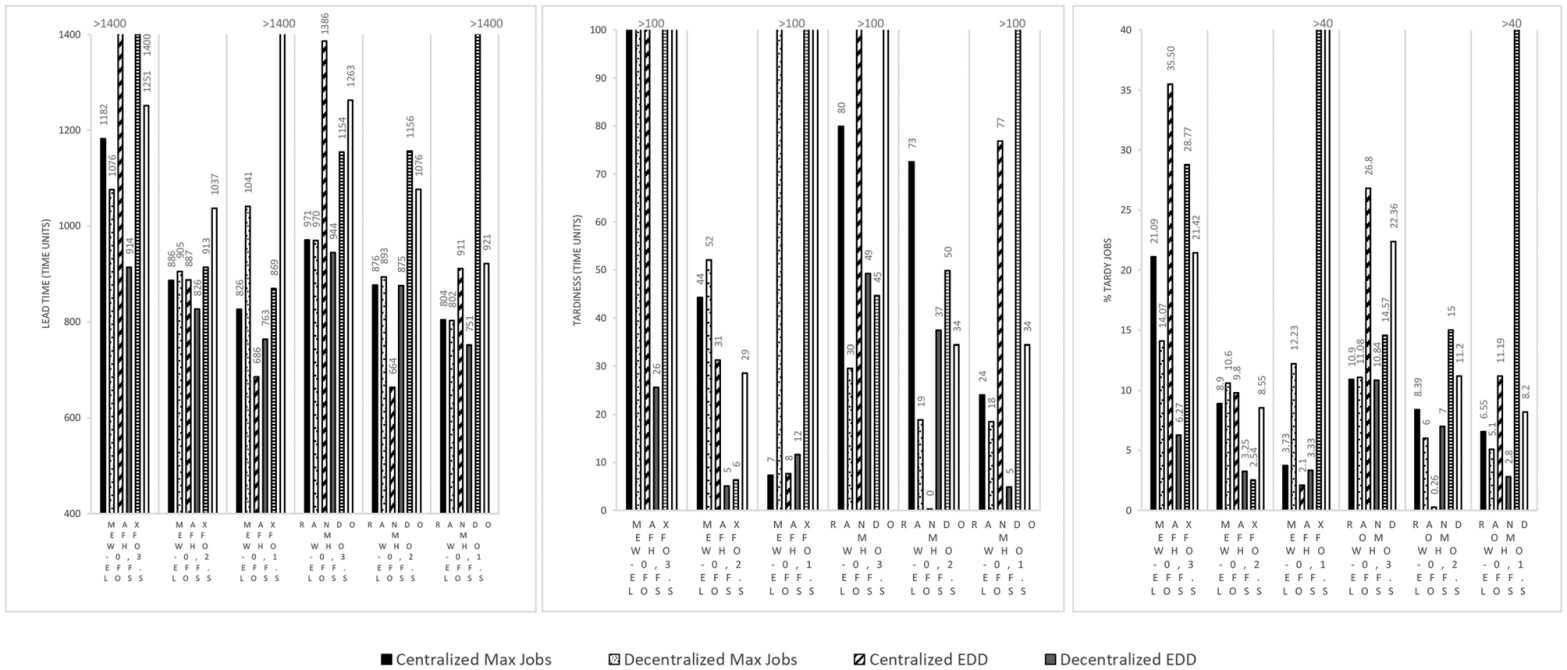
Lead time, Tardiness, and %Tardy Jobs for 2 Peaks configuration matrix

**Fig. 4:**
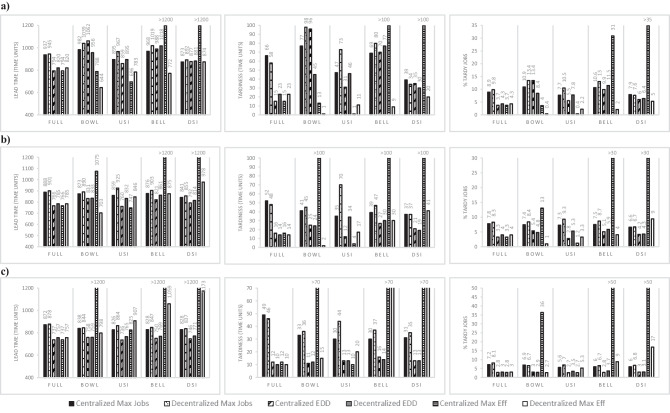
Lead time, Tardiness, and %Tardy Jobs for Efficiency with Random Who Rule for efficiency loss: (**a**) 0,3; (**b**) 0,2; and, (**c**) 0,1

Results in Fig. 2 highlight that with $\alpha$ equal to 0,3, in addition to Bowl, also USI and Bell perform better than Full while DSI performs similarly to Full. This means that when workers select the station where they are most efficient, configurations where most efficient workers are placed at the central or upstream stations outperform configurations where workers are all equally efficient or where most efficient workers are placed downstream. This raises the questions: What would happen if the most efficient workers are placed at the second and fourth station? Do the previous findings hold? To answer these questions, we designed a new matrix where the maximum average efficiency is at station 2 and 4, while the least efficient stations are stations 1, 3 and 5. This matrix creates “2 peaks”. It takes from Bowl that the first and last stations have the lowest efficiency, and from Bell that the central station has the lowest efficiency. Results in Fig. 3 show that this new matrix performs well with EDD, when it performs better than Bowl, Bell and USI, especially with a Max Eff Who rule and a Decentralized When rule. This is because there are more efficient workers on downstream stations, especially at station four. In accordance with previous results, the new matrix does not perform well with Max Jobs, especially with Decentralized and with Max Eff Where rule. This largely confirms our previous findings (Fig. 4) .

### Discussion of results

Our results confirm the DRC literature in that a heterogeneous workforce can perform similar to a homogeneous workforce in terms of multi-functionality and that a heterogeneous workforce can outperform a homogenous workforce in terms of efficiency (Bobrowski and Park 1993; Brusco and Johns 1998; Bokhorst et al. 2004a, b). It is further argued here that this is due to the experimental setting, being parameters typically adjusted in experiments related to efficiencies to create a fair comparison. Our results further confirm the literature on unbalanced lines in that placing more flexible workers at the middle stations is the best configuration (Shaaban and McNamara 2009). Our results extend this literature by showing that the bowl phenomenon is relevant in lines without limited inventory buffers if capacity is dual resource constrained. This is an important finding extending the applicability of the bowl phenomenon integrating two related but hitherto unconnected streams of literature. Moreover, results show that USI can outperform Bowl if combined with an EDD Where and a Centralized When Rule. In general, which matrix performs best is largely dependent on the assignment rules applied. There is a strong interaction between cross-training configuration and assignment rules. Another important implication is that the Where Rule appears to be more important in our study than the When Rule. This is in contrast to the general guideline in the DRC literature that the Where Rule has less of a performance impact than the When Rule (Xu et al. 2011). For example, USI and 2 Peaks should be combined with EDD, while DSI should be combined with Max Jobs Where rule.

In terms of assignment rules, our results confirm that EDD tends to move workers downstream (Thürer et al. 2019). At the same time, the Max Eff rule prevents workers to be shifted amongst stations. Both effects determine which matrices work best with these two rules, and which matrices should not be combined with these two rules. Bell, and DSI should not be combined with an EDD Where rule in the case of heterogeneous multi-functionality, USI should not be combined with Max Jobs while it performs well with EDD, and Long Chain should not be combined with Max Jobs. For heterogeneous efficiency, DSI and 2 Peaks should not be combined with a Max Eff Where rule; this is also valid for Bell, if combined with a Centralized When rule. Meanwhile, Bowl should not be combined with a Centralized Max Eff Where rule, especially if the efficiency loss is low. It performs however best with a Decentralized Max Eff Where rule. Finally, USI should be combined with a Max Eff Where rule or EDD, depending on the efficiency loss, DSI and Bell with Max Jobs and 2 Peaks with a EDD Where rule.

Finally, our results question Yue et al.’s (2008) argument that a Long Chain configuration outperforms a Small Chain since it allows for a better balance of the workload. This finding was obtained in a job shop where more than one worker can work at each station. In a pure flow shop, small chain may outperform long chain, unless the long chain is closed and a Centralized When and Max Jobs Where rule applied. The likelihood that downstream stations have a low utilization rate increases for Long Chain because worker 4 is shifted to work at station 3. Given the directed flow in the pure flow shop, this causes a build-up of the queue in front of station 4. This has important implications for future research and practice. If a directed flow is considered, it may be preferable to have some skills overlapping between some workers rather than having all workers with overlapping skills.

## Conclusions

A major aim of the DRC literature is to exploit labour flexibility by shifting workers from one station to another to improve throughput times and delivery related performance. In this context, a heterogeneous workforce can provide significant reduction in training needs and costs compared to a homogenous workforce. Heterogeneous labour, specifically in the context of DRC shops, has been modelled either in the form of a multi-functionality matrix, which indicates whether a worker can work at a station, or an efficiency matrix, which gives the efficiency of each worker at each station. However, these matrices are typically considered to be an environmental factor, and, to the best of our knowledge, existing research did not seek to design matrices with the objective to improve performance. In response, this study asked: *What is the best design for multi-functionality and efficiency matrices in a high variety make-to-order flow shop with heterogeneous labour?* This research advances existing DRC theory by showing: (i) which are the best designs for multi-functionality and efficiency matrices in terms of performance improvement in a DRC flow shop; and (ii) how the performances of those matrices are dependent on the When, Where and Who worker assignment rules. A major contribution of this study is to highlight the potential that lies in the conscious design of worker skill matrices. Specifically, a bowl-shaped design showed much promise. However which design to choose is contingent on the labour assignment rule applied. In general, results show that a heterogeneous workforce can realize performance that is comparable or even superior to a homogeneous workforce. This overcomes the trade-off between increases in flexibility and increases in cost. It allows to build resilient shops and supply chains cost efficiently, which is of utmost importance in the current uncertain business environment.

### Managerial implications

Our results show that lead time and delivery related performance can be improved if certain unbalances are created across stations. Our results answer the practical managerial question *How to best unbalance a DRC line?* Or better *How to cross-train workers on a line in order to maximise lead time and delivery related performances?* Managers can use the result of the present research to design training programs that create the best unbalances according to the worker assignment rules used. In fact, results also highlight the importance of choosing the right Where Rule in combination with a certain multi-functionality or efficiency configuration. For example, when workers are more trained to work at upstream stations, an EDD rule should be used, while, on the contrary, when workers are more trained to work on downstream stations, a Max Jobs rule should be used. It is of utmost importance, not to take the worker cross training decision without the decision on worker assignment rule that will be applied. Results also advise to prefer small chains of skills overlap over long chains of skills overlap in pure flow shops. This is different from findings for job shops and avoids low utilizations at downstream stations and the ensuing queue built up at those stations incurred otherwise.

### Limitations and future research

A main limitation of our study is that we have only considered one layout configuration, which is a DRC pure flow shop. Future research could consider different layout configurations, such as job shops. The experimental setting could also be extended by incorporating other environmental factors, such as different coefficients of variation for the processing times, or different degrees of due date tightness. We recognize these limitations, but we also consider our experimental design to be justified by the need to keep our study reasonably focused. Future research could also remove some of the simplifying assumption of the model, for example, by considering transfer time, or learning and forgetting effects of the workers. We considered worker efficiency and multi-functionality to be static in order to keep our study focused, but fatigue or forgetting are likely to create dynamic efficiencies in practice. Future research could assess how dynamic efficiencies effect our results. Finally, future research could also consider the impact of higher-level planning functions, such as order release rules, which focus on line balancing in terms of work content. The literature reports that the bowl phenomenon is even more important in context with limited buffer size.

## Data Availability

Data are made available on request.
